# Ecological Consistency of SSU rRNA-Based Operational Taxonomic Units at a Global Scale

**DOI:** 10.1371/journal.pcbi.1003594

**Published:** 2014-04-24

**Authors:** Thomas S. B. Schmidt, João F. Matias Rodrigues, Christian von Mering

**Affiliations:** Institute for Molecular Life Sciences and Swiss Institute of Bioinformatics, University of Zurich, Zürich, Switzerland; University of California Davis, United States of America

## Abstract

Operational Taxonomic Units (OTUs), usually defined as clusters of similar 16S/18S rRNA sequences, are the most widely used basic diversity units in large-scale characterizations of microbial communities. However, it remains unclear how well the various proposed OTU clustering algorithms approximate ‘true’ microbial taxa. Here, we explore the ecological consistency of OTUs – based on the assumption that, like true microbial taxa, they should show measurable habitat preferences (niche conservatism). In a global and comprehensive survey of available microbial sequence data, we systematically parse sequence annotations to obtain broad ecological descriptions of sampling sites. Based on these, we observe that sequence-based microbial OTUs generally show high levels of ecological consistency. However, different OTU clustering methods result in marked differences in the strength of this signal. Assuming that ecological consistency can serve as an objective external benchmark for cluster quality, we conclude that hierarchical complete linkage clustering, which provided the most ecologically consistent partitions, should be the default choice for OTU clustering. To our knowledge, this is the first approach to assess cluster quality using an external, biologically meaningful parameter as a benchmark, on a global scale.

## Author Summary

To characterize the composition of microbial communities, researchers often sequence and quantify specific marker genes, particularly the SSU (‘small subunit’) ribosomal RNA gene. One crucial step in such studies is the clustering of sequences into *Operational Taxonomic Units* (OTUs) of closely related organisms. However, this practice has repeatedly been called into question, arguing that the use of OTUs is not backed by microbial speciation theory. Here, we explore whether OTUs group ecologically similar organisms and show that indeed, OTUs are generally ecologically consistent. Moreover, we show how ecological consistency can be used as a measure of OTU ‘quality’ and compare different widely used OTU clustering methods. Our findings should help in the design and interpretation of SSU-based microbial ecology studies, in a research field that is only beginning to unfold its full potential to help understand life at the smallest scales.

## Introduction

Recent advances in sequencing technology have enabled researchers to characterize microbial diversity at previously unattainable scales. In large collaborative efforts, such as the Human Microbiome Project [1], selected environments have been probed to depths of millions of sequences, but even smaller-scale studies generate datasets of hundreds of thousands of reads. While providing great detail and resolution, datasets of such scopes pose a challenge to defining meaningful units of microbial diversity, and the choice of diversity unit definition may influence data analysis. Arguably, the gold standard for microbial diversity units are theory-informed definitions that would comply with a commonly accepted concept of bacterial speciation; in other words, operational units of diversity should approximate ‘true’ bacterial taxa [2]. This implies two frequently cited criteria for theory-compliant diversity units: they should reflect phylogeny (by representing *monophyletic* groups of organisms) and ecology, since ecological differentiation has been postulated as an important driver of bacterial speciation [2]–[8]. However, a unifying concept of bacterial speciation in fact remains controversial to the point of contesting the very existence of ‘bacterial species’ as such [2], [9]–[11]. Nevertheless, approaches towards reconciling diversity unit definitions with evolutionary theory have received much attention. For example, the ecotype model of bacterial speciation defines basic diversity units as ecologically coherent groups of organisms whose diversity is confined by a cohesive genetic force [3], [12], and dedicated algorithms have been developed to demarcate ecotypes from environmental sequencing data [4]. However, while ecotype simulation has been valuable in characterizing the diversity of selected environments [13], it has been noted that recognized diversity clusters within several microbial clades can conflict with ecotype theory [11], [14].

Given the lack of a commonly accepted bacterial species concept, a phenomenological (pragmatic) approach to categorizing microbial diversity is often chosen in practice: *Operational Taxonomic Units* (OTUs), defined as clusters of 16S/18S small subunit (*SSU*) rRNA gene similarity, are used as theory-agnostic approximations of microbial taxa. Providing impartial partitions of complex sequence datasets, OTUs are the backbone of established workflows for the ecological characterization of microbial communities, such as *mothur* [15] or *QIIME* [16]. Several methods have been developed for binning SSU sequences, most prominently *hierarchical clustering algorithms* (*HCA*, implemented e.g. in *mothur*) and their heuristic approximations, such as *uclust* [17], *cd-hit* [18] or the *ESPRIT* suite of algorithms [19], [20]. However, it has been noted that different clustering methods often provide highly inequivalent partitions of the same data, both quantitatively (with respect to total cluster counts and OTU size distributions) and qualitatively (with respect to cluster composition) [21]–[24]. Consequently, several studies have evaluated approaches to SSU clustering, focusing on distinct measures of cluster quality. Probably the most straightforward test for OTU partition quality has been the comparison of total OTU counts between methods, based on simulated or experimental samples of known composition [19], [21], [24], [25]. Schloss & Westcott [22] used Matthew's Correlation Coefficient as an internal measure of partition quality, based on cluster composition. Alternatively, methods have been benchmarked against taxonomically typed ground truth partitions, using measures such as *Variation of Information* (*VI*, [26]), *Normalized Mutual Information* (*NMI*, [20], [23], [24]) or cluster *Purity* [24] to assess taxonomic consistency. This optimization for taxonomically ‘pure’ clusters is attractive under the assumption that taxonomic consistency implies both phylogenetic and ecological consistency. However, existing taxon delineations may frequently conflict with phylogeny or refer to ecologically heterogeneous groups of organisms [10], and conflicts between available reference taxonomies, as well as database bias, further reduce the indicative power of taxonomic labels when describing broad ranges of microbial diversity. Moreover, it has been shown that both *NMI* and *VI* produce shifting baseline values, depending on the number of clusters investigated [27], an effect that none of the above-mentioned studies corrects for. Finally, relying on simulated or experimental mixes of known composition as defined inputs may run the danger of missing fundamental challenges brought on by real-world samples (such as micro-heterogeneity, long-tailed abundance distributions, cellular debris, chimeric molecules, contaminations, etc.). Thus, while taxonomic ‘ground truth’ may often give a reasonable first assessment, what are alternative and more generally applicable parameters for characterizing ‘good’ basic units of diversity in microbial ecology?

In this study, we explore the ecological consistency of OTUs. We first revisit and confirm the observation that ecological preferences of microbial lineages are deeply rooted in phylogeny: organisms that share a high SSU sequence similarity tend to be ecologically more similar than expected by chance. We then explore whether this signal is captured by SSU-based OTUs: do organisms that cluster into the same OTU share similar ecological affiliations? In other words, are OTUs ecologically consistent? We approach these questions by first providing anecdotal evidence, before then introducing an *Ecological Consistency Score (ECS)* to provide a more thorough evaluation of OTU ecological consistency. Using a global dataset of roughly one million near full-length SSU sequences, we compare different widely used methods for SSU clustering with respect to how ecologically consistent the OTUs are that they generate. Finally, we reflect on the validity and usefulness of SSU-based OTUs as fundamental units of microbial diversity in light of their ecological consistency, and discuss the implications of using ecological consistency as a taxonomy-independent measure of clustering quality.

## Methods

### Sequence data & preprocessing

To obtain a comprehensive global dataset, we extracted all full-length 16S/18S rRNA sequences from NCBI *GenBank* ([28], accessed in April 2012) and from the genomes available in the NCBI Reference Sequence Database (*RefSeq* [29], accessed in March 2012). After using Infernal to align sequences to reference consensus models of the bacterial, archaeal and eukaryotic 16S/18S rRNA molecules (provided in the package *ssu-align* [30], [31]) and after removing ∼20% of total reads that were flagged as chimeric by UCHIME [32], we pruned away any terminal nucleotides that aligned outside of two manually chosen, well-conserved start- and end-positions in the alignment. After these steps, our dataset comprised 950,014 aligned, near full-length sequences (see Text S1 for details).

### Sequence clustering into Operational Taxonomic Units

We clustered sequences into OTUs using three HCAs (*average, complete and single linkage*) and two heuristic methods (*uclust, cd-hit*). For every method, we clustered to thresholds of 80–99% sequence identity (92–99% for *average linkage*, see Text S1). We generated OTU sets using *cd-hit* ([18], version 4.5.4, Build 2012-08-25) in *cdhit-est* mode (recommended for clustering highly similar sequences) using standard parameters. The *uclust* ([17], http://drive5.com/usearch/, version 6.0.307) series of OTU sets was generated using the *uclust* software with the *cluster*_*fast* option and standard parameters. Hierarchical *average*, *complete* and *single linkage* clustering were implemented using the recently developed in-house software package *hpc-clust* [33] using the ‘*onegap*’ sequence distance calculator (counting gaps as single mismatches). *Hpc-clust* parallelizes the hierarchical clustering task and has been shown to cluster sequences as fast as, or even faster than heuristic implementations such as *uclust* and *cd-hit* (less than 3 h wall time for the present dataset of roughly one million sequences on a 256 core computer cluster), while still computing the entire pairwise distance matrix, avoiding any heuristic shortcuts.

### Contextual data

We extracted different types of ecologically relevant information from *GenBank* and *RefSeq* annotations. First, we assigned sequences to individual *sampling events* that we define here as unique combination of submitting authors, publication title and isolation source; this classified the dataset into 31,519 samples. Next, we filtered free-text annotations down to 7,202 unique, non-trivial ecological *terms* describing the sampling context. Using a manually curated classification scheme, we annotated samples to 53 more broadly defined *habitat types* (e.g., ‘skin’ or ‘soil’, see Text S1 for the full list). In a complementary approach, we filtered annotation keywords for the controlled vocabulary maintained by the Environmental Ontology Project (*EnvO*, http://environmentontology.org/, release date 2011-24-03) and used the ontology to assign related environmental terms to samples (e.g., ‘lake’ and ‘pond’ were both classified as ‘water body’). This procedure yielded 672 unique *EnvO terms* represented in the dataset. Finally, for samples that are associated with a eukaryotic host, we assigned *host taxonomy* from direct annotations and by inference from annotation keywords. This procedure yielded 2,422 unique host taxonomies (in total representing 5,850 unique taxa) represented in the dataset; remaining archaeal and bacterial sequences were considered *non host-associated*.

### Assessing global-scale ecological consistency of OTUs

We developed an *Ecological Consistency Score (ECS)* to assess the ecological consistency of entire sets of sequence clusters with respect to different ecological signals (such as *ecological terms*, see above). The *ECS* was calculated as follows. Consider a partition of a SSU sequence dataset into *N* OTUs of sizes *n_1_, n_2_, …, n_N_*. What is the likelihood that an ecological feature *j* with a background frequency of *p_j_* in the entire dataset is observed exactly *k_i,j_* times in OTU *i* of size *n_i_*? We calculated this likelihood *L_i,j_* using a binomial model:
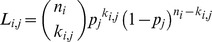



For example, observing 5 sequences annotated with the ecological term ‘skin’ (background frequency of 30.0%) in an OTU containing 15 sequences has a likelihood of 0.206, but observing the much less frequent term ‘hydrothermal’ (background frequency ∼0.9%) exactly 5 times in the same OTU is much less likely (*L_15,hydrothermal_* = 1.6*10^−7^). Similarly, *not* observing a frequent term such as ‘skin’ in the same OTU has a rather low likelihood (*L_15,skin_* = 0.005). Thus, the presence of 5 sequences annotated as ‘hydrothermal’ in an OTU of size 15 is an *enrichment of ecologically similar organisms*, while the absence of a frequent term such as ‘skin’ in the same OTU is a *negative enrichment*. We computed the summed log-likelihood *LL_set_* of the entire partition from the enrichment of every term *j* in every OTU *i*:
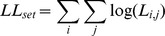



High absolute values of *LL_set_* indicated that the distribution of ecological features across the various OTUs in the entire partition were non-random. However, the absolute value of *LL_set_* is influenced by total OTU count (as the number of summands *i*) and OTU size distribution (as *n_i_* in the binomial coefficient). We used an empirical approach to control for these effects: we computed the log-likelihoods *LL_rand_* of 1,000 randomized sets with identical cluster size distribution and total count, but with shuffled sequence-to-OTU mapping. This generated a (near-Gaussian) background distribution of *LL_rand_*, from which we calculated the *ECS* of the observed OTU set as standard Z score:
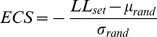
where *μ_rand_* is the average value of *LL_rand_* and *σ_rand_* is the standard deviation. Thus, *ECS* values indicate by how many standard deviations the enrichment of ecological features in the observed OTU set is removed from a randomized background. In other words, the *ECS* indicates how consistent a given set of OTUs is with respect to an ecological signal, such as the distribution of ecological terms.

## Results

### SSU similarity is indicative of ecological similarity, and vice versa

**Figure 1:**
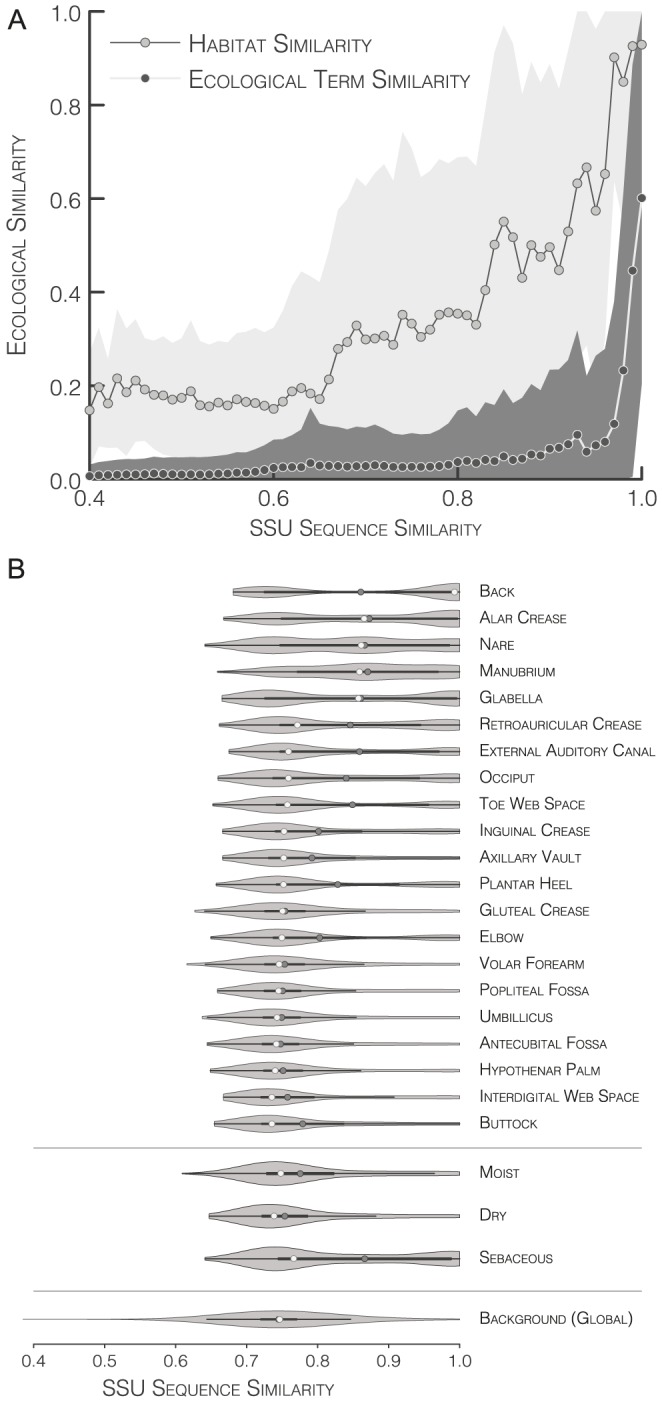
Phylogenetic similarity vs. ecological similarity. (A) General correspondence of ecological and SSU similarity. From our global dataset of roughly one million SSU sequences, 20 datasets of 10,000 sequences each were randomly sampled. For each subset, pairwise sequence similarities and ecological similarities (as *Jaccard Index* of shared annotated ecological terms and habitat types, respectively) were calculated, and the results were averaged over the 20 sets before plotting; mean standard deviations across sets are indicated by grey shades. (B) Internal 16S SSU similarity of human skin habitats. For the *human skin microbiome* dataset [37], pairwise SSU similarities were calculated for all sequences sampled from respective human skin habitats (top) and for sequences from habitats of the same type (‘moist’, ‘dry’ or ‘sebaceous’, as classified by Grice et al [37]; middle). Global background similarities were obtained by calculating pairwise internal SSU similarities for 20 sets of 10,000 sequences randomly drawn from our environmentally heterogeneous set of roughly one million SSU sequences (bottom). Smoothened distributions were drawn based on 150,000 randomly sampled pairwise distances. White circles indicate median, grey circles mean similarity. Non-smoothened, detailed distributions are available in Figures S1 and S2.

Several recent studies have shown that microbes can be remarkably *niche conservative*: ecological affiliations such as habitat preferences are rooted deeply in the tree of life [34], [35]. As a consequence of this ‘ecological coherence of high bacterial taxa’, a close relationship between ecological similarity and SSU similarity has been observed. We confirmed this relationship by exploring a novel, global sequence dataset of roughly one million near full-length SSU sequences, for which we automatically inferred sampling habitats based on ecologically relevant annotation keywords. We calculated pairwise similarities in SSU sequences, ecological terms and inferred habitats (as Jaccard index) for 20 sets of 10,000 randomly selected sequences, resulting in a total of ∼10^9^ pairwise comparisons; the results are shown in Figure 1A. For both ecological terms and inferred habitats, we observed a clear trend towards higher ecological similarity at higher SSU similarity. This observation is in line with previous studies that reported a very similar pattern of increasing ecological similarity with decreasing distance on SSU-based phylogenetic trees [34], [36]. Moreover, it is concordant with general niche conservatism in microbes, given that our dataset represents a diverse and global survey of microbial taxa. In other words, phylogenetic distance is indicative of ecological similarity. But is the reverse also true? Are ecologically coherent groups of organisms more similar in SSU sequence similarity than expected by chance?

To assess the *internal* SSU similarity of ecologically coherent groups of organisms, we reanalyzed the *human skin microbiome* (HSM) dataset that provides ∼100,000 near full-length 16S sequences sampled from distinct body sites [37]. Considering each body site as a unique habitat, we calculated pairwise 16S sequence similarities per sample; the results are shown in Figure 1B, Figures S1, S2 and Table S1. All habitats showed a major abundance of sequence pairs in the 70–80% 16S similarity range, likely corresponding to comparisons of organisms from different bacterial phyla. However, several habitats showed distinctly bimodal (e.g. back, toe web space) or multimodal (e.g. nare, manubrium) distributions of internal 16S similarities, indicating an abundance of more closely related organisms (Figure 1B, top panel). Indeed, these observations are in line with the habitat-wise diversity estimates provided in the original HSM study [37]. When compared to a global background dataset of bacterial 16S sequences (Figure 1B, bottom panel), all skin habitats showed both a notable overrepresentation of highly similar sequence pairs (>90% 16S similarity), as well as the complete absence of a ‘tail’ of highly dissimilar pairs (<60% 16S similarity). In other words, organisms sampled from a defined skin habitat were more similar to each other in 16S sequence than expected for a global background; this enrichment was statistically highly significant (p<<10^−16^, one-sided Mann-Whitney-U test, see Table S1). The same was true for more broadly defined habitat types: 16S sequences sampled from ‘moist’, ‘dry’ and ‘sebaceous’ skin sites (as classified in the original HSM study) shared significantly higher similarity than expected for a background set (Figure 1B, middle panel, Figure S2 and Table S1). This indicates that in spite of local diversity and distinct internal 16S similarity profiles, the different ecologically coherent habitats (body sites, skin habitat types) sustained communities containing more closely related organisms (higher 16S similarity) than expected for a global background.

Taken together, these results confirm a close relationship between ecological and SSU similarity: closely related organisms tend to be ecologically more similar than expected by chance. However, the reverse is also true: ecological similarity is often indicative of increased SSU similarity.

### OTUs are ecologically homogenous on a broad ecological scale

How does this relation between ecological and SSU similarity translate to Operational Taxonomic Units? Are clusters defined by SSU similarity ecologically consistent? To approach these questions, we clustered a global dataset of roughly one million SSU sequences into OTUs according to different methods that implement fundamentally different clustering regimes. *Hierarchical Clustering Algorithms (HCAs)* compute an entire matrix of pairwise sequence distances and progressively merge the most similar clusters, while *heuristic* methods provide computationally efficient shortcuts. The *complete linkage* (*cl*, *furthest neighbor*) HCA implements an *exclusive* clustering regime, joining two clusters only if every pairwise similarity between the members of each cluster is above the clustering threshold. In contrast, *single linkage* (*sl*, *nearest neighbor*) is *inclusive*, as clusters are joined as soon as any two of their members share above-threshold similarity. Average linkage (*al*, *average neighbor* or *unweighted pair group method with arithmetic mean, UPGMA*) conceptually provides a middle ground between the two, requiring that the average pairwise similarity between all members of two clusters be above the threshold for joining them. The most widely employed *heuristic* methods for SSU sequence clustering are arguably *uclust* [17] and *cd-hit* [18]. *Uclust* defines cluster seed sequences, usually depending on sequence length or abundance in the dataset, to which sequences are subsequently compared and linked if the similarity (computed as number of shared short ‘words’, or *k-mers* between the sequences) is above the required threshold; note that in consequence, *uclust* combines the three steps of sequence alignment, alignment distance calculation and clustering into one. Similarly, *cd-hit* assigns sequences to representative cluster seeds, but uses a different word-matching algorithm and replaces (even implicit) sequence alignment altogether by the use of indexing tables.

**Figure 2:**
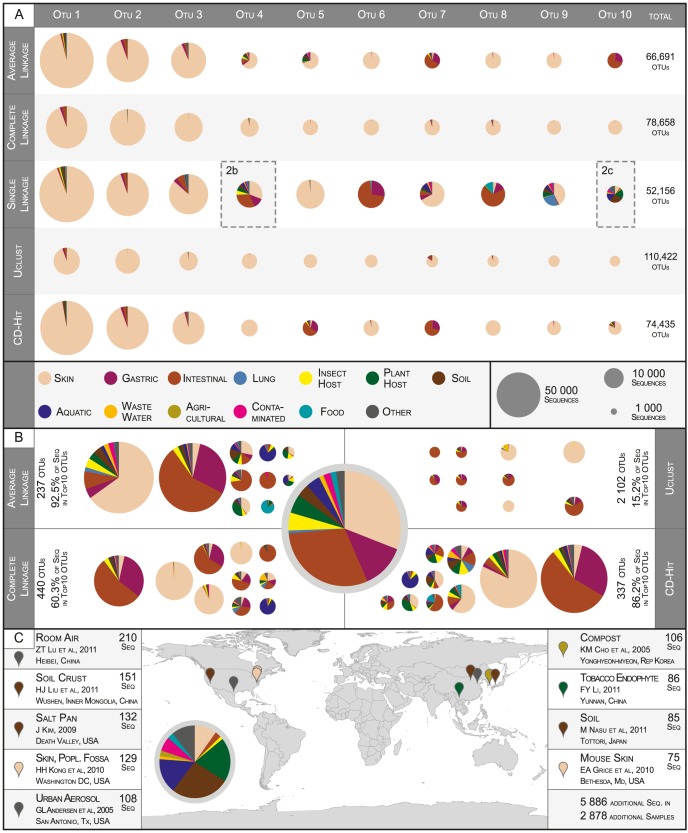
Broad-scale ecological homogeneity of OTUs. (A) Habitat associations of the ten largest OTUs when clustering a comprehensive set of publicly available full-length SSU sequences to 97% similarity using different methods. Pie chart area is proportional to OTU size, colors correspond to habitat types. Total OTU counts are indicated on the right. 9.7% of publicly available sequences lacked habitat annotation, or were typed to conflicting habitats, and were excluded from the analysis. Note that the OTUs shown are not generally identical across clustering methods, but overlap in sequence composition. (B) Breaking down the ecologically inconsistent cluster ‘*sl* OTU 4’. In the presence of the full global dataset, different methods cluster the 17,462 sequences in *sl* OTU 4 differently, mostly providing ecologically more homogeneous clusters. For every method, the ten largest clusters and the fraction of sequences they contain, as well as total OTU counts are shown. (C) Sampling events contributing to ‘*sl* OTU 10’. Geographic locations and isolation sources are shown for nine of the largest sampling events. Marker colors indicate habitat type.

Figure 2A shows the ecological associations of the ten largest OTUs for every method when clustering to 97% SSU sequence similarity. We observed that for all methods except *sl*, the majority of OTUs was ecologically homogenous. Clearly, the dominating habitat in the overall dataset, skin (30% of total sequences), also dominated most of the ten largest OTUs for every method, with gastric and intestinal habitats as the second most important fraction. In particular for *cl* and *uclust*, all studied OTUs except ‘*uclust* OTU 7’ consisted of ≥95% sequences sampled from skin, and almost all remaining sequences in these OTUs were annotated as gastric or intestinal. Similarly, most of the observed *al* and *cd-hit* OTUs were dominated by these habitats, albeit to lower extent and with notable exceptions (*al* OTUs 4 & 7, *cd-hit* OTU 5). In contrast, *sl* produced several large clusters that were ecologically heterogenous (OTUs 4, 7–10), with the dominant habitat representing as little as 26.6% of sequences in *sl* OTU 10.

Figure 2B provides a closer look at *sl* OTU 4. It consisted of 17,462 habitat-typed sequences of highly diverse ecological affiliation; for example, sequences sampled from insect hosts, plant hosts, aquatic environments or soil each accounted for 4–5% of diversity within this OTU. We observed that all other tested methods generated significantly more OTUs from the same 17,462 sequences when clustering in the context of the full global set of roughly one million sequences. Indeed, the observed differences in total OTU counts were in the range of 2–3 orders of magnitude, with *uclust* providing 2,102 OTUs where *sl* provided only one. At the same time, we observed that both *cl* and *uclust* provided ecologically more homogenous partitions of the same sequence set, notably by distributing sequences associated to skin and to gastric/intestinal habitats largely into distinct OTUs. Likewise, *al* and *cd-hit* provided ecologically more consistent OTUs than *sl*, albeit to lesser extent. Although all four methods also generated several ecologically heterogenous OTUs, their overall partitions appeared ecologically more homogenous than the single ecologically inconsistent cluster generated by *sl*.

As another example, consider the largest sampling events contributing to *sl* OTU 10 (Figure 2C). Clearly, this OTU contained sequences from very distinct and unrelated ecological contexts, not only on the level of broad habitat types (skin, soil, etc.), but also at finer ecological resolution (e.g., different soil types). Interestingly, this ecological heterogeneity corresponded to a large internal SSU dissimilarity of this particular OTU: although clustered to a nominal similarity threshold of 97%, we observed that a large majority of pairwise similarities within *sl* OTU 10 were actually below this threshold (as can be expected for an *inclusive* clustering algorithm), at a mean internal similarity of 95.2% and with individual pairs of sequences sharing as little as 86% SSU similarity.

The above observations are mostly anecdotal: we considered only a small selection of OTUs and elaborated on individual examples. Nevertheless, this may help to illustrate two important points that will be discussed more rigorously in the following sections: (i) the tested methods clustered the same sequence dataset very differently with respect to total OTU count, OTU size distribution and OTU ecological homogeneity; (ii) with the exception of *sl*, clusters were generally homogenous on a broad ecological scale, considering e.g. that skin and gastric/intestinal habitats are arguably more similar to each other than they are to aquatic or soil habitats.

### Global-scale ecological consistency of OTUs depends on clustering method

To refine our above observations on general OTU ecological homogeneity, we developed an *Ecological Consistency Score* (*ECS*, see Methods). Adopting a global perspective rather than focusing on individual examples, the *ECS* is a measure of ecological consistency of entire OTU partitions, taking into account all the clusters provided by a given clustering method. Moreover, focusing on more fine-scale ecological associations than provided by the broadly defined habitat types discussed above, the *ECS* provides increased ecological resolution. High *ECS* values indicate that ecologically similar organisms are clustered, more so than expected by chance.

**Figure 3:**
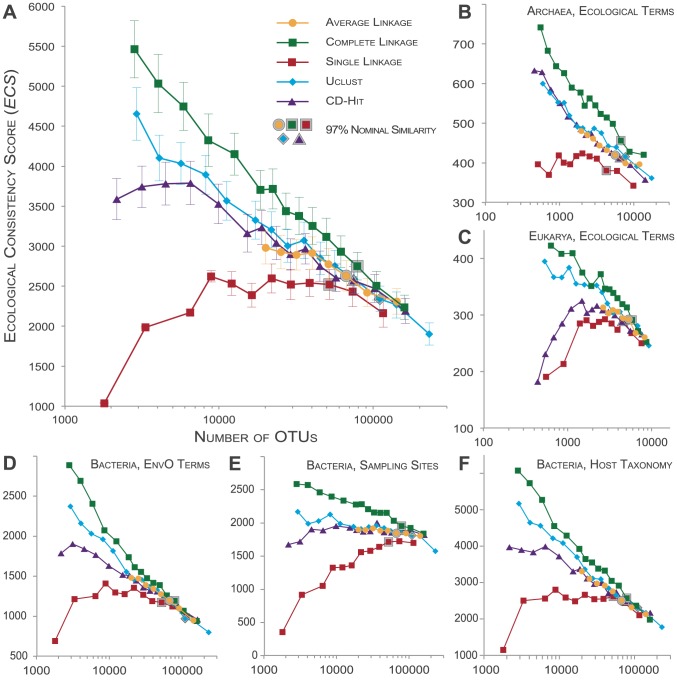
Global *Ecological Consistency Scores* of OTUs. (A) Ecological term consistency when clustering 887,870 *bacterial* full-length 16S sequences according to different methods. *ECS* values (y-axis) describe how non-random the enrichment of ecological affiliations is in a given OTU set (see main text). The total number of clusters including singletons (x-axis) provides for better comparability of methods than nominal clustering thresholds; lower numbers of OTUs correspond to less stringent similarity cutoffs. Error bars indicate jackknifed estimates of *ECS* variability (see Text S1). Data points for OTU sets clustered to 97% nominal sequence similarity are highlighted with a grey shade. The raw data are available in Table S2. For the ecological term consistency when clustering 42,402 archaeal sequences (B), or 20,120 eukaryotic 18S sequences (C), as well as for the bacterial dataset EnvO term consistency (D), sampling site consistency (E), and host taxonomy consistency (F), error bars are not drawn, but variability was in the same range as for (A) (coefficients of variation, 0.06<c_V_<0.08).

We tested cluster consistency with respect to four distinct ecological signals: (i) 7,202 *ecological terms* (Figure 3A–C), which we filtered from sequence annotations, provided detailed descriptions of sampling context; (ii) 672 *EnvO terms* (Figure 3D), which we filtered from annotation keywords using the EnvO ontology, provided an alternative and curated hierarchy of ecological descriptions; (iii) *sampling site information* (Figure 3E), for which we considered whether a given OTU contained many sequences that had been sampled from the same site; and (iv) *host taxonomy* (Figure 3F), assuming that closely related host organisms generally provide more similar environments than more distantly related ones. We processed these signals independently, calculating an *ECS* for a given OTU partition for each ecological signal.

We calculated the *ECS* for OTU sets obtained from clustering our global set of roughly one million sequences to nominal similarity thresholds of 80%–99% (92%–99% for *al*, see Text S1) according to different methods: *al*, *cl*, *sl*, *uclust* and *cd-hit* (Figure 3 and Table S2). For all tested datasets, and over the entire range of tested OTU set sizes, we observed similar trends in ecological consistency (*ECS* from highest to lowest): *cl*, *uclust*, *cd-hit*/*al* and *sl*. Over wide ranges of tested OTU counts, differences between OTU definitions were statistically significant (one-sided t-test on jackknifed estimate of *ECS* variability, p<<0.01). Jackknifed *ECS* variability was low and constant for all tested datasets and OTU set sizes (coefficient of variation, 0.06<c_V_<0.08).

We observed different and reproducible trends in *ECS* within clustering methods. With increasing clustering stringency (increasing similarity threshold, increasing number of total clusters), *ECS* values monotonically decreased for *cl*, *uclust* and *al*, and for *cd-hit* in the high-cutoff range. This general decrease in ecological consistency might indicate that the rather broad ecological descriptions aligned better with OTUs at lower nominal similarity thresholds, while more closely defined OTUs (higher cluster counts) were not equally well resolved on an ecological scale. In contrast, we observed the opposite trend (decreasing *ECS* with decreasing stringency) for *sl*, and to a lesser extent sometimes *cd-hit*, at lower clustering thresholds. As *sl* is an *inclusive* algorithm (see above), it tends to cluster sequences that share below-threshold similarity. For example, in the previous section we pointed out ‘*sl* OTU 10’, the 10th largest *sl* OTU when clustering to 97% similarity, which clustered sequences sharing below-threshold similarity (mean internal similarity of 95.2%, most dissimilar sequence pair sharing 86% similarity). Since such lumping behavior aggravates with decreasing clustering stringency, it may explain the observed decrease in ecological consistency.

*ECS* differences between methods were more pronounced with increasing levels of clustering: while at very high similarity thresholds (≥99%), partitions were similar and sometimes indistinguishable on an *ECS* scale, differences of up to ∼5-fold between *cl* and *sl* were observed at lower sequence similarity levels. At the frequently-used similarity threshold of 97%, *ECS* scores of *cl* were between 10% and 20% higher than those of *sl*, depending on the feature tested (Table S2). *Cl* also consistently showed the highest *ECS* values when the set of SSU sequences was restricted to those from completed sequenced genomes only (Figure S3). Distinct ecological signals provided different levels of *ECS* resolution: at higher OTU counts, keyword-based measures were less distinctive on an *ECS* scale (ecological term consistency, Figure 3A, and EnvO term consistency, Figure 3D), while sampling site consistency separated OTU definitions better (Figure 3E). Likewise, the archaeal sequence dataset (Figure 3B) distinguished different OTU definitions better than the larger bacterial (Figure 3A) and smaller eukaryal (3C) datasets. However, the general trend was the same across all tested datasets, and across all indicators of ecological consistency: *complete linkage (cl)* generated ecologically more consistent OTUs than the other methods; *single linkage (sl)* resulted in the lowest *ECS* values in all tests; and the remaining methods fell into an intermediate range, while *uclust* generally provided higher ecological consistency than *cd-hit* and *al* which in turn were mostly indistinguishable from each other.

## Discussion

### Ecological consistency of OTUs is a matter of perspective

Are SSU-based OTUs ecologically consistent? Our results indicate that they are, to a large extent. We detected high levels of ecological consistency both at broad ecological scale in individual examples (Figure 2) and at finer ecological scale for a global SSU dataset (Figure 3). In contrast, Koeppel and Wu [13] recently reported an ‘extensive ecological heterogeneity among OTUs’ for very fine-scale habitat definitions of two model datasets of marine *Vibrio* [5] and hot spring *Synechococcus* [38] communities. Thus, OTU ecological consistency may in fact be a matter of perspective: while OTU clustering may conflict with very high-resolution ecological associations for specific environments, OTUs are generally, though not perfectly, consistent on broader ecological scales. Considering that OTU clustering is a phenomenological approach to diversity analysis, the observed levels of ecological consistency are remarkable: although OTU definitions are mostly independent of underlying assumptions on microbial ecology, they capture groupings of ecologically coherent organisms.

Are the observed levels of ecological consistency sufficient for OTUs to be useful in the ecological characterization of microbial communities? Indeed, it is difficult to globally define appropriate levels of required ecological consistency for ‘good’ units of microbial diversity. This is largely due to the *ecological plasticity* of microbial taxa at different levels of taxonomic and ecological resolution: while broad-scale ecological coherence in general is deeply rooted in phylogeny [34], several cases of wide ‘intra-species’ ecological variation have been reported, e.g. within the genera *Bacillus* [39] or *Escherichia* [40]. In other words, though relatedness at family, order or even phylum level is often predictive of a common broad ecological niche, very closely related lineages frequently exhibit surprisingly wide ecological differentiation.

Another frequently cited criterion for biologically meaningful basic diversity units is *phylogenetic consistency*. While Koeppel and Wu recently reported ‘extensive and pronounced paraphyly and polyphyly among OTUs’ when compared with the *ecotype simulation* algorithm (which uses a phylogenetic tree as input, [13]), we found surprisingly high levels of phylogenetic coherence of *complete linkage* OTUs: with respect to a maximum likelihood tree of 42,024 archaeal sequences, >80% of all non-singleton OTUs at different clustering thresholds were monophyletic (Text S2).

In general, conceptually more sophisticated algorithms to demarcate OTUs such as *ecotype simulation* [4], *CROP* [41] or *M-Pick* [42] may be suited for focused problems, but arguably suffer from throughput problems due to high computational demands (we were not able to execute any of them on our set of one million sequences). On the other hand, impartial OTU clustering conquers large and complex datasets rapidly, while still providing reasonably high levels of ecological consistency. For in-depth studies on broader ecological scopes, OTUs may thus provide good approximations of ecologically coherent lineages.

### How good is ‘good enough’? Ecological consistency and cluster quality

While we found that OTUs are ecologically consistent in general, there were significant differences between clustering methods. Are these differential levels of ecological consistency indicative of clustering quality? We have shown that an ecological similarity signal, calculated based on contextual data alone, corresponds to SSU similarity for a global, environmentally heterogenous dataset, as well as for the well-defined *human skin microbiome* dataset (Figure 1). Based on this observation, high internal SSU similarity in microbial diversity clusters is expected to correspond to high ecological consistency. In other words, metadata-based ecological consistency can provide a non sequence-based, external measure of cluster quality. Moreover, it is arguably useful to consider ecological consistency when evaluating the quality of diversity units in the context of microbial ecology; nevertheless, ecologically *plastic* diversity units should not be considered inherently ‘wrong’, since ecological differentiation may occur within groups of closely related organisms. The *Ecological Consistency Score* casts these ideas into an objective framework; it is a global measure of ecological consistency for entire partitions of microbial diversity datasets. Several previous approaches to assessing clustering quality relied on measures such as *Normalized Mutual Information* or *Variation of Information*; these can be problematic, as they are biased by variation in total cluster counts and cluster size distributions [27]. Correcting for these effects, *ECS* values are comparable between different diversity unit definitions.

Considering that our dataset provides a comprehensive survey of microbial diversity, the observed differences in ecological consistency have several interesting implications when interpreted in terms of cluster quality. The tested methods implement different assumptions on the fundamental organization of microbial diversity. Conceptually, *sl* clustering is *inclusive* (guaranteeing that all pairs of above-threshold similarity are clustered, tending to provide fewer and large clusters), while *cl*, *uclust* and *cd-hit* are *exclusive* (preventing any below-threshold pair from clustering and thus tending to provide smaller and more compact clusters); *al*, which focuses on average similarity, provides a balanced middle ground. Our results indicate that exclusive clustering regimes, and in particular *cl*, provide ecologically much more consistent partitions than the inclusive regime of *sl*, and somewhat surprisingly also than *al*. While exclusive and inclusive regimes by definition may provide different partitions at the same nominal similarity threshold in terms of cluster counts, sizes and composition, *ECS* values correct for these effects, in particular when compared across partitions of similar total cluster counts rather than similar nominal sequence similarity. We note that the most rigidly exclusive clustering regime, *uclust*, which at any given threshold provided significantly more (and smaller) OTUs than all other methods, did not provide the highest *ECS* values, probably indicating an over-partitioning of ecologically homogenous clusters.

One potential pitfall of our dataset is sampling bias: clearly, a comprehensive survey of available SSU data will be ‘anthropocentric’, since in the past, sequencing efforts have been disproportionally concentrated on the human microbiome; for example, ‘skin’ was the overall most frequent ecological term in the set, annotated to as many as 30% of all sequences. However, the *ECS* framework corrects for potential impacts of this sampling bias by providing the exact same input sequences for each tested method, by using weighted background frequencies for every ecological feature, and by randomizing partitions conservatively. Indeed, our dataset meets many characteristics of reference datasets for *reference-based* approaches to OTU demarcation, as implemented e.g. in QIIME [16]. Such approaches rely on well-defined, comprehensive and usually pre-clustered sets of reference sequences that serve as a ‘backbone’ to guide the mapping and OTU binning of novel reads. Consequently, the choice of reference pre-clustering method can have a strong impact on resulting reference-based picked OTUs; some of the most commonly used reference sets, provided by the Greengenes [43] and SILVA [44] databases, rely on *uclust* for pre-clustering. As ecological consistency can be an important parameter to optimize for in such globally applicable reference sets, our results may inform the choice of pre-clustering method in such contexts.

Finally, as our findings pertain to global taxonomic and ecological scopes, they are of potential interest for the ongoing debate between taxonomic ‘lumpers’ and ‘splitters’ [45]–[47], considering that exclusive clustering corresponds to ‘splitting’ regimes, while ‘lumping’ is inclusive.

When designing a workflow to analyze large sequence datasets, informed choices of methods and parameters are needed at many levels. For example, different denoising protocols, filters for chimeric sequences and alignment methods have previously been benchmarked and are not within the scope of our study. Here, we have focused on sequence clustering into OTUs, and our results may contribute to a more informed choice of clustering method when studying microbial communities: of all tested methods, *complete linkage (cl)* may provide the ecologically most consistent partitions of large sequence datasets. Moreover, there are clearly other aspects of clustering quality that we have not touched upon here, such as robustness to the choice of sequenced SSU gene subregion, portability across studies or the impact of dataset context (does a given method cluster ‘rich’ and ‘sparse’ datasets differently?). Nevertheless, ecological consistency is an important parameter to optimize for, in particular when later using OTUs for the ecological characterization of microbial communities.

To our knowledge, our study provides the first benchmark for SSU clustering methods that employs a signal *external* to both taxonomy and sequence. As more and more environments become available to in-depth ecological characterization, it will be interesting to explore alternative paths towards adopting ecology not only into species concepts, but also into definitions of microbial diversity units. Indeed, our results suggest that ‘traditional’ OTU clustering has yet an important role to play in this process.

## Supporting Information

Figure S1 **Pairwise sequence similarities within** ***human skin microbiome*** **habitats.** This figure contains un-smoothened versions of the sequence similarity distributions shown in Figure 1B. Pairwise internal sequence similarity distributions are shown for every skin habitat from the HSM dataset. Background similarities (indicated in grey) were calculated from 20 sets of 10,000 sequences which were randomly drawn from the global set of bacterial 16S sequences. All similarities were calculated using *hpc-clust* [33].
(PDF)

Figure S2 **Sequence similarities within human skin microbiome habitat types.** Skin habitats were classified into three types (‘moist’, ‘dry’, ‘sebaceous’) in the original publication by Grice et al [37]. In the upper panel, this figure shows un-smoothened versions of the sequence similarity distributions shown in the middle panel of Figure 1B. Pairwise sequence similarities within habitat types were plotted against similarities between sequences drawn from the global background set (indicated in grey; see Figure S1).
(PDF)

Figure S3 **Ecological consistency of OTUs from 4,485 16S gene sequences from fully sequenced genomes.** We extracted 4,485 16S genes from fully sequenced genomes downloaded from the RefSeq database [29] and clustered them into OTUs according to different methods (see Methods section in the main text). *ECS* values for all five tested methods are shown; partitions at 97% nominal sequence similarity are highlighted with a grey shade.
(PDF)

Table S1 **Sequence similarities within** ***human skin microbiome*** **subsets.** This table provides the main statistics on sequence similarities for all tested HSM habitats, habitat types and the global background set (Fig. 1B, S1, S2). The rightmost column indicates the p value for a one-sided unpaired Mann-Whitney-U-test of the type ‘*internal sequence similarity within habitat X is greater than background similarity’*. To calculate internal similarities for the different habitat types (indicated by a star, ‘*’), 10,000 sequences were randomly drawn from the full sets per habitat type.
(XLSX)

Table S2 **Ecological term consistency of clustering methods across similarity thresholds when clustering 887.870 bacterial sequences.** 887.870 bacterial sequences were clustered using the hierarchical clustering algorithms *average linkage*, *complete linkage* and *single linkage* (implemented in *hpc-clust*, [33]) and the heuristics *uclust* and *cd-hit*. An Ecological Consistency Score (*ECS*) was calculated with respect to filtered ecological annotation terms. The table reports total OTU counts and ECS values (mean and jack-knifed standard deviation, see Methods in main text); the data corresponds to that shown in Figure 3A in the main text.
(XLSX)

Text S1 **Supplementary Methods.**
(PDF)

Text S2 **Phylogenetic consistency of OTUs.** For a global dataset of 42,024 archaeal sequences, *complete linkage* OTUs were tested for monophyly with regard to a maximum likelihood phylogenetic tree.
(PDF)
