# Supplementary figures and images for: Ecological Consistency of SSU rRNA-Based Operational Taxonomic Units at a Global Scale
Source: PLoS Comput Biol. 2014 Apr 24;10(4):e1003594. doi: 10.1371/journal.pcbi.1003594 (PMC3998914; doi:10.1371/journal.pcbi.1003594)

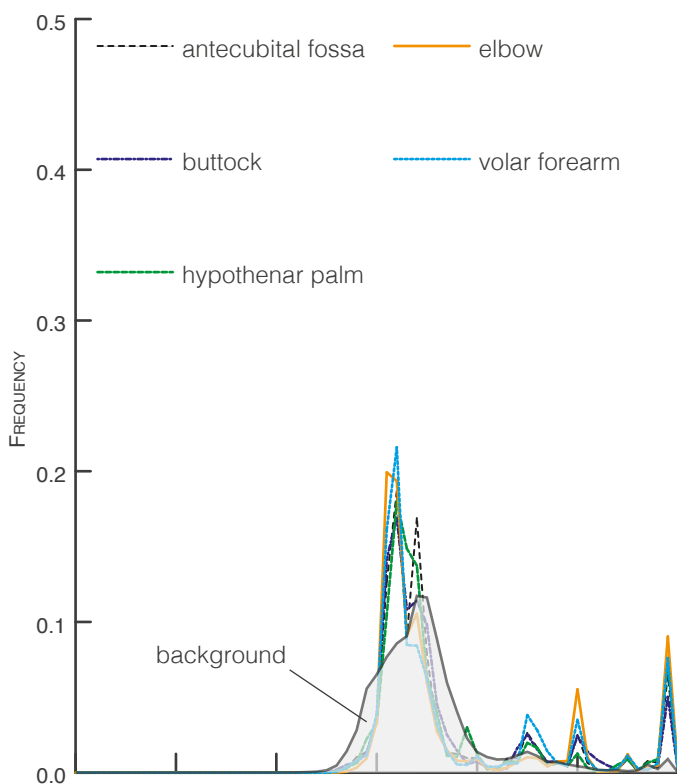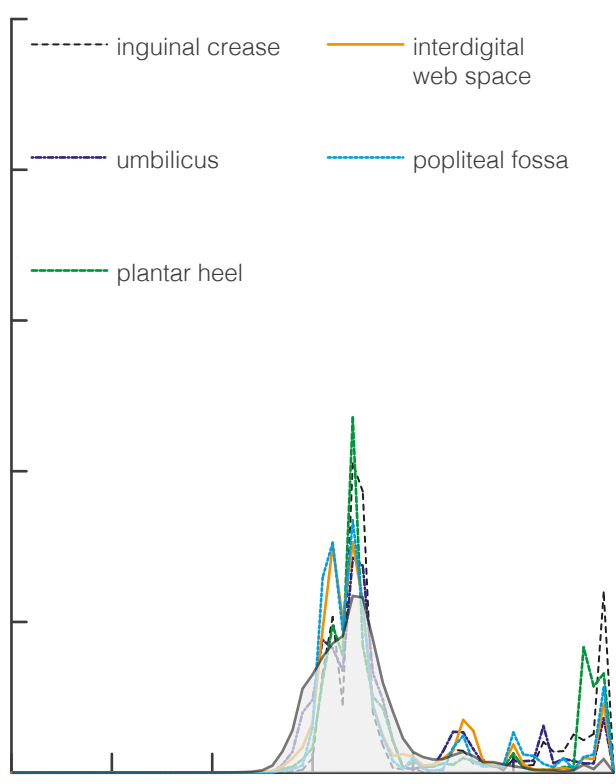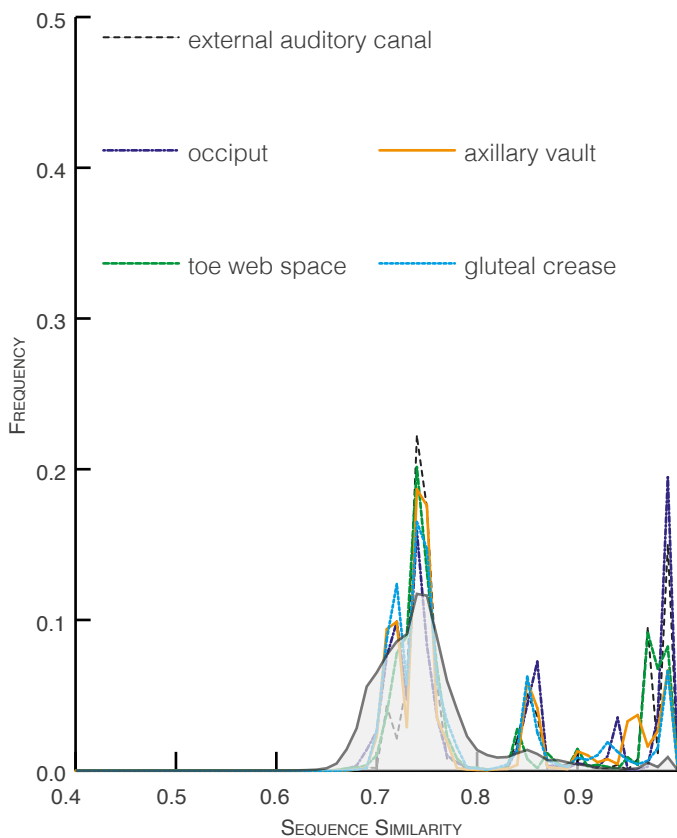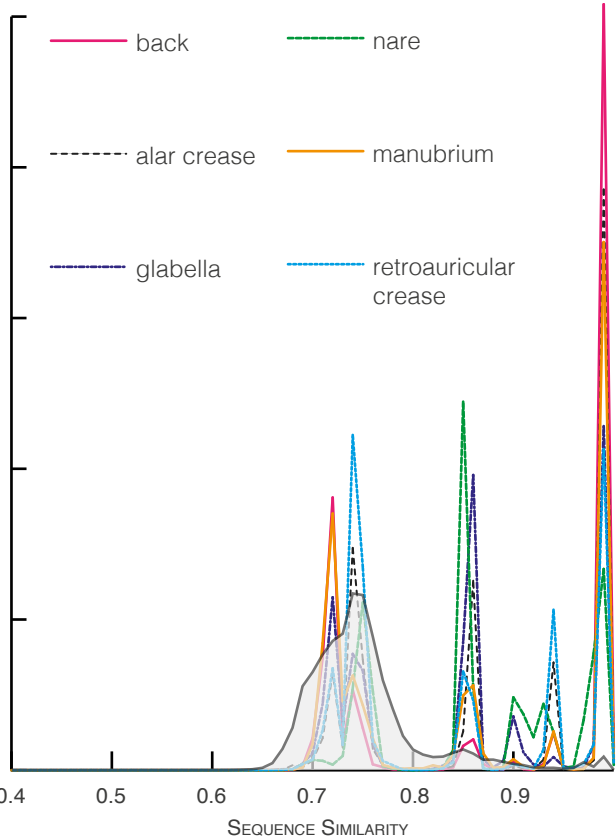

Supplement: Figure S1 — Pairwise sequence similarities within human skin microbiome habitats. This figure contains un-smoothened versions of the sequence similarity distributions shown in Figure 1B. Pairwise internal sequence similarity distributions are shown for every skin habitat from the HSM dataset. Background similarities (indicated in grey) were calculated from 20 sets of 10,000 sequences which were randomly drawn from the global set of bacterial 16S sequences. All similarities were calculated using hpc-clust [33]. (PDF) [file pcbi.1003594.s001.pdf]

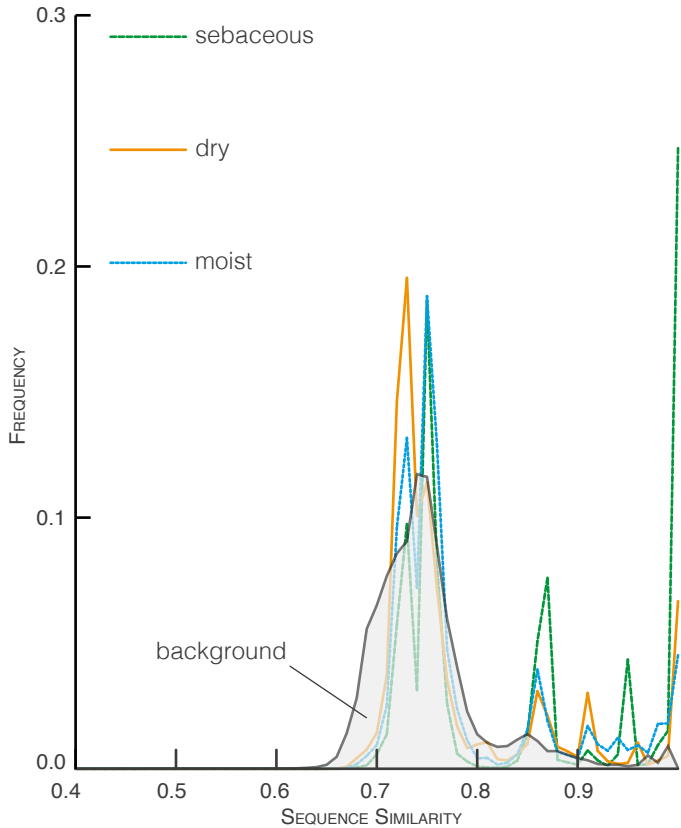

Supplement: Figure S2 — Sequence similarities within human skin microbiome habitat types. Skin habitats were classified into three types (‘moist’, ‘dry’, ‘sebaceous’) in the original publication by Grice et al [37]. In the upper panel, this figure shows un-smoothened versions of the sequence similarity distributions shown in the middle panel of Figure 1B. Pairwise sequence similarities within habitat types were plotted against similarities between sequences drawn from the global background set (indicated in grey; see Figure S1). (PDF) [file pcbi.1003594.s002.pdf]

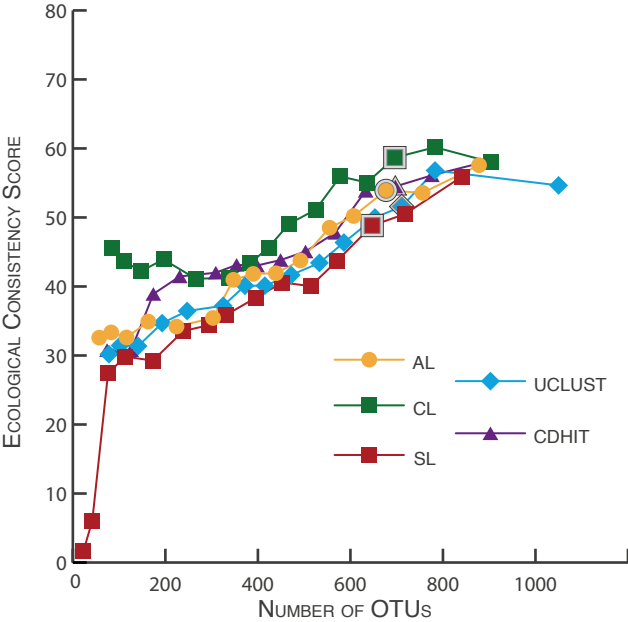

Supplement: Figure S3 — Ecological consistency of OTUs from 4,485 16S gene sequences from fully sequenced genomes. We extracted 4,485 16S genes from fully sequenced genomes downloaded from the RefSeq database [29] and clustered them into OTUs according to different methods (see Methods section in the main text). ECS values for all five tested methods are shown; partitions at 97% nominal sequence similarity are highlighted with a grey shade. (PDF) [file pcbi.1003594.s003.pdf]
