# Supplementary material for: Ecological Consistency of SSU rRNA-Based Operational Taxonomic Units at a Global Scale
Source: PLoS Comput Biol. 2014 Apr 24;10(4):e1003594. doi: 10.1371/journal.pcbi.1003594 (PMC3998914; doi:10.1371/journal.pcbi.1003594)
Supplement: Text S1 — Supplementary Methods. (PDF) [file pcbi.1003594.s006.pdf]

Sequence Data & Preprocessing

SSU rRNA sequences were downloaded from NCBI *GenBank* ([1], http://www.ncbi.nlm.nih.gov/genbank/, accessed in April 2012) and from the genomes available in the NCBI Reference Sequence Database (*RefSeq*, [2], http://www.ncbi.nlm.nih.gov/RefSeq/, accessed in March 2012). From these sources, we filtered for sequences that were annotated as 'ribosomal RNA' or 'rRNA' and had a minimum length of 1,000bp.

We generated a pseudo-multiple sequence alignment (pseudo-MSA) of our entire dataset from pairwise alignments of sequences to curated covariance models using the alignment software *Infernal* [3]. *Infernal* provides very fast and accurate profile-based alignments that take into account the SSU RNA molecule's highly specific secondary structure. We aligned all sequences to reference consensus models of the bacterial and archaeal 16S rRNA molecule and the eukaryotic 18S rRNA molecule as provided in the package *ssu-align* ([3,4], http://infernal.janelia.org). In a recent study, Wang et al found that for the alignment of SSU sequences, structure-aware approaches such as used by *Infernal* did not outperform traditional alignment methods, such as the Needleman-Wunsch algorithm [5]. However, Wang et al used a dataset of relatively short sequences (231bp) from the V2 region of the SSU molecule which exhibits relatively little secondary conformation. Moreover, to assess alignment quality, they used a NMI metric to test accordance with a 'ground truth' dataset; this approach is questionable for this particular kind of problem, as discussed in the main text. Moreover, Schloss has pointed out a series of further limitations in the Wang et al commentary and discussed the use of secondary structure informed alignment methods [6]. In using full-length sequences that have on average a much higher degree of structural information than the V2 region only, we are confident that a structure-aware approach adds accuracy to our alignments.

We assigned sequences to the three phylogenetic domains of life (archaea, bacteria and eukarya) based on which reference model they aligned to with the highest *Infernal* alignment score; sequences with a negative score for all three models were excluded from the analysis altogether. To obtain an alignment of uniform length, comprising the same amount of information for every sequence, we pruned all sequences at manually chosen flanking positions (alignment positions 142 to 899 for the archaeal model, 107 to 1,408 for bacteria, and 629 to 1,547 for eukarya), yielding three distinct alignments of lengths 757bp, 1,301bp and 918bp, respectively. We filtered for chimeric sequences using UCHIME [7] with a set of reference sequences generated *de novo* from the entire alignments. This way, 18.9%, 19.7% and 9.7% of the sequences were identified as chimeric and removed for subsequent analysis. After these pre-processing steps, the dataset used in this study comprised 950,014 sequences (42,024 archaeal, 887,870 bacterial and 20,120 eukaryotic) of which 720,086 or 75.8% were unique (30,962, 673,128 and 15,996, respectively). These sequences each cover (approximately) the entire 16S/18S SSU rRNA molecule.

Sequence Clustering into Operational Taxonomic Units

We clustered sequences into OTUs using several established approaches: we executed both heuristic methods (*uclust*, *cd-hit*) and hierarchical clustering algorithms (HCA; *average, complete* and *single linkage*). For every applied method, we clustered to different sequence identity thresholds (80, 82, 84, 86, 88, 90, 91, 92, 93, 94, 95, 96, 97, 98 and 99 percent SSU similarity).

We generated OTU sets using *cd-hit* ([8,9], http://weizhong-lab.ucsd.edu/cd-hit/, version 4.5.4, Build 2012-08-25) in *cdhit-est* mode (recommended for clustering highly similar sequences) on a multicore machine using parallelization and standard parameters and word length 11. We tested word lengths of 7, 9 and 11 as parameters in the calculation of sequence similarity; however, while longer word lengths provided significant speed improvements, the observed differences both in OTU total count and size distribution, as well as in ecological consistency of the resulting OTU sets were negligible (data not shown) so that we discuss only results for word length 11. The *uclust* ([10], http://drive5.com/usearch/, version 6.0.307) series of OTU sets was generated using the *uclust* software with the *cluster_fast* option and standard parameters.

Hierarchical *average, complete* and *single linkage* clustering was implemented using our recently developed in-house software package *hpc-clust* [11]. While *cl* and *sl* partitions were obtained for the whole range of tested similarity thresholds, *al* clustering of the large bacterial dataset was only performed for ≥92% 16S similarity clusters due to high memory requirements of the algorithm. *Hpc-clust* parallelizes the hierarchical clustering task and thus allows to cluster large datasets very rapidly (less than 3h wall time for the present dataset of roughly one million sequences on a 256 core computer cluster), while still computing the entire pairwise distance matrix, avoiding any heuristic shortcuts. Moreover, the software provides the option to use different *alignment distance* calculation functions; however, since the OTU sets generated by different tested distance calculation methods showed no significant differences in ecological consistency (data not shown), we present only results obtained using the *onegap* alignment distance calculator, counting gaps of any length between sequences as single mismatches.

Finally, we attempted to cluster the sequence dataset with the commonly used software tools *mothur* ([12], version 1.27.0, 2012-08-08) and *ESPRIT-Tree* ([13], version 1, 2011-11-15). However, we were unable to process the entire dataset of roughly one million full-length sequences, or even smaller subsets of ≥100k sequences with either of these programs, even when providing excessive computational resources (running on a multicore computer with 1TB RAM); this is most likely due to the computationally expensive calculation of the pairwise SSU sequence distance matrix.

Contextual Data

Both the *GenBank* and *RefSeq* databases provide facilities for submitting rich metadata with each sequence. We harvested this contextual information in several ways to get a description of ecological properties of the organisms represented by the SSU rRNA sequences in the present dataset. First, we assigned sequences to individual *sampling events* that we define here as a unique combination of submitting authors, publication title and isolation source; this classified the dataset into 31,519 samples, the largest of which comprised 61,479 sequences, at an average sample size of 30.2 sequences per sample.

Next, we extracted *annotation keywords* for every sample from the publication title, isolation source and additional comments (GenBank annotation field 'note'). We filtered these keywords by removing any terminal letters 's' (to map plural forms) and by requiring that in order to be valid, a keyword had to be used by at least two author teams independently. In addition, we filtered for (potentially misleading) taxonomic and geographic annotations by removing all keywords that produced a hit in the NCBI *Taxonomy* database ([14], http://www.ncbi.nlm.nih.gov/taxonomy) or the *GeoNames* database of geographical place names (http://www.geonames.org). Moreover, we removed keywords that clearly carried no information ecologically characterizing a sample (such as the word 'DNA') using a manually curated list of 1,144 stop words. In total, these filtering steps reduced the number of annotation keywords by roughly one order of magnitude, yielding 7,202 unique *ecological terms*, at an average frequency of 18.76 samples per term. The vast majority of these terms carry biological information characterizing SSU sequences with respect to the ecological and environmental context in which they were sampled. Based on these ecological terms and on host organism annotations (see below), we annotated samples to a list of 53 unique *habitat types* using a manually curated classification scheme (see Table 1). Habitat typing was non-exclusive: individual samples could be annotated with different habitat subtypes, e.g. 'aquatic, marine, benthic' or 'forest soil, rhizosphere'.

In a complementary approach, we filtered all keywords for the controlled vocabulary maintained by the Environmental Ontology Project (*EnvO*, http://environmentontology.org/, release date 2011-24-03) and used the ontology to assign related environmental terms to sequences (e.g., 'lake' and 'pond' were both classified as 'water body'). This procedure yielded 672 unique *EnvO terms* mapping to 16,736 samples – indicating that nearly half of all samples could not be annotated using EnvO. However, having been derived using a dedicated ontology for environmental terms, these keywords carry much ecological information.

Finally, we assigned *host taxonomy* to bacterial and archaeal sequences from direct annotations (*GenBank* annotation field 'host') and by inference from annotation keywords (terms matching the NCBI Taxonomy that mapped to higher plants or metazoans were considered to refer to putative host organisms). This yielded 2,422 unique host taxonomies (in total representing 5,850 unique taxonomic categories) for a total of 9,621 samples; the remaining 21,898 samples were considered *not host-associated*. The by far most highly represented host organism was *Homo sapiens* (407,107 sequences in 1,003 samples); in general, animal hosts (572,675 sequences) were much more represented than plant hosts (30,210).

Habitat Type	Habitat Subtype	# of SSU Sequences
anthropogenic	contaminated*	45,990
	wastewater	14,445
	food (fermented)	2,333
	food (dairy)	3,419
	food (other)	32,889
	sterile	3,492
	agricultural	41,348
	other (anthropog.)	29,687
	total (anthropog.)	127,491
host-associated	plant (phyllosphere)	2,197
	plant (rhizosphere)	5,576
	plant (other)	58,095
	skin	342,533
	gastric	63,580
	intestinal	182,003
	oral	3,254
	lung	7,064
	vaginal	803
	blood	1,815
	human host	502,955
	mammalian host	534,766
	insect host	20,166
	animal host (other)	598,106
	total (host-ass.)	643,613

Habitat Type	Habitat Subtype	# of SSU Sequences
aquatic	marine	64,648
	limnic	21,889
	estuarine	3,836
	littoral	21,274
	pelagic	8,429
	benthic	44,184
	lake	16,459
	stream	6,153
	ice	3,042
	saline	7,806
	other	145,056
	aquatic (total)	230,691
terrestrial (soil)	arctic	2,223
	arid	2,319
	cave	7,382
	forest	3,627
	grassland	11,160
	wetland	9,469
	rock & mineral	14,260
	total (soil)	120,534
thermal	hydrothermal	10,138
	geothermal	12,200
	total (thermal)	19,680
unclassified	total	41,260

Table 1: Habitat Classification of 950,457 SSU sequences. Habitat typing was non-exclusive: sequences could be associated with multiple habitat (sub-)types. For example, marine aquatic environments could be further classified as benthic, pelagic or littoral; samples from the gastrointestinal tract were annotated as both 'gastric' and 'intestinal', etc. Note also that host-associated habitats were assigned based on both annotation terms and annotated host information.

*Contaminated habitats were classified into additional subgroups: oil, heavy metal, metal, radioactive and polyaromatic hydrocarbon contamination.

Ecological Consistency of OTUs

We developed an *Ecological Consistency Score (ECS)* to assess the ecological consistency of entire sets of sequence clusters with respect to different ecological signals.

Consider an individual OTU *i* clustering n_i SSU sequences from different sampling events. Each sequence is annotated according to different ecological signals characterizing the environment from which it was sampled, such as e.g. *ecological terms* or *host organism taxonomy*. We consider an OTU *ecologically consistent* if it is enriched in sequences that share similar ecological affiliations. We calculated the likelihood $L_{i,j}$ of observing any ecological feature *j* (e.g., an ecological term such as 'soil', 'skin' or 'ocean') with a global background frequency p_j in the entire dataset exactly $k_{i,j}$ times in an OTU *i* of size n_i using a binomial model:

$$L_{i,j} = \binom{n_i}{k_{i,j}} p_j^{\,k_{i,j}} \left(1 - p_j\right)^{n_i - k_{i,j}}$$

For example, observing 5 sequences annotated with the ecological term 'skin' (background frequency of 30.0%) in an OTU containing 15 sequences has a likelihood of 0.206, but observing the much less frequent term 'hydrothermal' (background frequency ~0.9%) exactly 5 times in the same OTU is much less likely ($L_{15,hydrothermal} = 1.6*10^{-7}$). Similarly, not observing a frequent term such as 'skin' in the same OTU has a rather low likelihood ($L_{15,skin} = 0.005$). Thus, the presence of 5 sequences annotated as 'hydrothermal' in an OTU of size 15 is an *enrichment of ecologically similar organisms*, while the absence of a frequent term such as 'skin' in the same OTU is a *negative enrichment*.

While $L_{i,j}$ describes the ecological consistency of an individual OTU, what is the likelihood of the enrichment of *all* ecological features across *all* sequence clusters in the dataset? We computed this as the summed log-likelihood LL_{set} over all $L_{i,j}$:

$$LL_{set} = \sum_i \sum_j \log(L_{i,j})$$

High absolute values of LL_{set} indicate that enrichments of ecological features in OTUs across the entire partition are non-random. However, the absolute value of LL_{set} is influenced by total OTU count (as the number of summands *i*) and OTU size distribution (as n_i in the binomial coefficient). Thus, in order to compare biological consistency between OTU sets, we used an empirical approach to control for these effects. For any given OTU set, we generated 1,000 randomized sets with identical OTU size distribution, but shuffled sequence-to-OTU mapping and computed the summed log-likelihood LL_{rand} for each of these sets. This generated near-Gaussian distributions of randomized set log-likelihoods LL_{rand}. From this, we calculated the biological consistency score of the observed OTU set as standard Z score:

$$ECS = -\frac{LL_{set} - \mu_{rand}}{\sigma_{rand}}$$

where μ_{rand} is the average value of LL_{rand} and σ_{rand} is the standard deviation. Thus, *ECS* values indicate by how many standard deviations the enrichment of ecological features in an observed OTU set is removed from a randomized background. The *ECS* controls for both OTU size distribution effects and total number of OTUs in the set and provides a measure that is comparable between OTU sets.

An empirical jack-knifing approach was used to assess *ECS* variability. Based on the 1,000 randomized LL_{rand} values, *ECS* was recalculated 1,000 times from 100 randomly drawn values for every OTU set. From the resulting *ECS* distributions, *ECS* variability (as *coefficient of variation*) and statistical significance of *ECS* differences (using one-sided Student's tests) were calculated.

References

1. Benson DA, Cavanaugh M, Clark K, Karsch-Mizrachi I, Lipman DJ, et al. (2013) GenBank. Nucleic Acids Research 41: D36–D42. doi:10.1093/nar/gks1195.
2. Pruitt KD, Tatusova T, Brown GR, Maglott DR (2011) NCBI Reference Sequences (RefSeq): current status, new features and genome annotation policy. Nucleic Acids Research 40: D130–D135. doi:10.1093/nar/gkr1079.
3. Nawrocki EP, Kolbe DL, Eddy SR (2009) Infernal 1.0: inference of RNA alignments. Bioinformatics (Oxford, England) 25: 1335–1337. doi:10.1093/bioinformatics/btp157.
4. Nawrocki EP (2009) Structural RNA Homology Search and Alignment Using Covariance Models. Saint Louis (Missouri): Washington University in Saint Louis, School of Medicine. Available: http://openscholarship.wustl.edu/etd/256/.
5. Wang X, Cai Y, Sun Y, Knight R, Mai V (2011) Secondary structure information does not improve OTU assignment for partial 16s rRNA sequences. ISME J 6: 1277–1280. doi:10.1038/ismej.2011.187.
6. Schloss PD (2012) Secondary structure improves OTU assignments of 16S rRNA gene sequences. ISME J 7: 457–460. doi:10.1038/ismej.2012.102.
7. Edgar RC, Haas BJ, Clemente JC, Quince C, Knight R (2011) UCHIME improves sensitivity and speed of chimera detection. Bioinformatics (Oxford, England) 27: 2194–2200. doi:10.1093/bioinformatics/btr381.
8. Li W, Godzik A (2006) Cd-hit: a fast program for clustering and comparing large sets of protein or nucleotide sequences. Bioinformatics (Oxford, England) 22: 1658–1659. doi:10.1093/bioinformatics/btl158.
9. Fu L, Niu B, Zhu Z, Wu S, Li W (2012) CD-HIT: accelerated for clustering the next-generation sequencing data. Bioinformatics (Oxford, England) 28: 3150–3152. doi:10.1093/bioinformatics/bts565.
10. Edgar RC (2010) Search and clustering orders of magnitude faster than BLAST. Bioinformatics (Oxford, England) 26: 2460–2461.
11. Rodrigues JFM, Mering CV (2013) HPC-CLUST: Distributed hierarchical clustering for very large sets of nucleotide sequences. Bioinformatics (Oxford, England). doi:10.1093/bioinformatics/btt657.
12. Schloss PD, Westcott SL, Rabyn T, Hall JR, Hartmann M, et al. (2009) Introducing mothur: Open-Source, Platform-Independent, Community-Supported Software for Describing and Comparing Microbial Communities. Appl Environ Microbiol 75: 7537. doi:10.1128/AEM.01541-09.
13. Cai Y, Sun Y (2011) ESPRIT-Tree: hierarchical clustering analysis of millions of 16S rRNA pyrosequences in quasilinear computational time. Nucleic Acids Research 39: e95. doi:10.1093/nar/gkr349.
14. Federhen S (2012) The NCBI Taxonomy database. Nucleic Acids Research 40: D136–D143. doi:10.1093/nar/gkr1178.
