# Supplementary material for: Ecological Consistency of SSU rRNA-Based Operational Taxonomic Units at a Global Scale
Source: PLoS Comput Biol. 2014 Apr 24;10(4):e1003594. doi: 10.1371/journal.pcbi.1003594 (PMC3998914; doi:10.1371/journal.pcbi.1003594)
Supplement: Text S2 — Phylogenetic consistency of OTUs. For a global dataset of 42,024 archaeal sequences, complete linkage OTUs were tested for monophyly with regard to a maximum likelihood phylogenetic tree. (PDF) [file pcbi.1003594.s007.pdf]

Phylogenetic consistency is a frequently cited criterion for biologically meaningful basic units of microbial diversity [1-3]. In this view, 'good' diversity unit definitions, complying with evolutionary theory, should provide clusters which are *monophyletic*, rather than *paraphyletic* or *polyphyletic*.

To assess the phylogenetic consistency of OTUs, we analyzed a global dataset of 42,024 near-full length archaeal 16S sequences (see main text and Text S1). We generated a Maximum Likelihood tree assuming a generalized time-reversible (GTR) model using FastTree2 [4]. To this tree, we mapped OTUs obtained from *complete linkage* clustering to different similarity thresholds. As a measure of phylogenetic consistency, we calculated an OTU *monophyly index* with respect to the reference tree. We considered an individual OTU as '100% monophyletic' if (i) all its members shared a single common ancestor, and (ii) no members of the same monophyletic group clustered with any other OTU. To account for different patterns of paraphyly or polyphyly in individual OTUs, we defined P_{anc} as the most recent common ancestor of all sequences pertaining to that OTU. We then calculated 'local monophyly' of the focal OTU as the ratio of sequences belonging to the focal OTU (N_{OTU}) relative to all sequences descending from the most recent common ancestor P_{anc} (N_{desc}) in an approach similar to Koeppel & Wu [5]. For example, an OTU containing 9 sequences which form a paraphyletic group with one additional sequence clustered to another OTU was considered '90% monophyletic'. The overall monophyly index for an entire OTU set was then calculated as the average of the local monophyly of non-singleton OTUs. Note that singleton OTUs (containing only one sequence) are monophyletic by definition, and were not considered when calculating average monophyly, but could locally break monophyly within larger OTUs.

We observed a monophyly index of around 80% for clustering thresholds ≥84% sequence similarity (see data table). These levels of monophyly are remarkably high, in particular when considering that the reference tree itself is probably a close approximation, rather than perfect representation, of the 'true' phylogeny of the tested dataset. We conclude that *complete linkage* hierarchically clustered OTUs are generally, though not perfectly, phylogenetically consistent.

**% Sequence Similarity**	80	82	84	86	88	90	92	94	96	98	99
**Total number of clusters**	589	745	958	1,200	1,525	1,973	2,644	3,677	5,381	9,160	13,685
**Non-singleton clusters**	327	419	544	671	868	1,067	1,392	1,835	2,548	3,670	4,470
**Monophyly Index in %**	73.1	74.7	80.4	79.7	80.4	80.6	80.3	81.1	82.1	81.3	80.1

Table 1: Phylogenetic Consistency of *complete linkage* OTUs. 42,024 archaeal 16S sequences were clustered into OTUs and monophyly was assessed with respect to a maximum likelihood phylogenetic tree as described in the text.

References

1. Gevers D, Cohan FM, Lawrence JG, Spratt BG, Coenye T, et al. (2005) Re-evaluating prokaryotic species. Nat Rev Microbiol 3: 733–739. doi:doi:10.1038/nrmicro1236.

2. Koeppel A, Perry EB, Sikorski J, Krizanc D, Warner A, et al. (2008) Identifying the fundamental units of bacterial diversity: a paradigm shift to incorporate ecology into bacterial systematics. Proc Natl Acad Sci USA 105: 2504–2509. doi:10.1073/pnas.0712205105.

3. Fraser C, Alm EJ, Polz MF, Spratt BG, Hanage WP (2009) The bacterial species challenge: making sense of genetic and ecological diversity. Science 323: 741–746. doi:10.1126/science.1159388.

4. Price MN, Dehal PS, Arkin AP (2010) FastTree 2 – Approximately Maximum-Likelihood Trees for Large Alignments. PLOS ONE 5: e9490. doi:10.1371/journal.pone.0009490.s003.

5. Koeppel AF, Wu M (2013) Surprisingly extensive mixed phylogenetic and ecological signals among bacterial Operational Taxonomic Units. Nucleic Acids Research 41: 5175–5188. doi:10.1093/nar/gkt241.
